# Do the presence, size, and shape of plantar calcaneal spurs have any significance in terms of pain and treatment outcomes in patients with plantar fasciitis?

**DOI:** 10.55730/1300-0144.5598

**Published:** 2022-11-26

**Authors:** Mehmet OKÇU, Figen TUNCAY, Fatmanur Aybala KOÇAK, Yakup ERDEN, Mustafa Yemliha AYHAN, Samet Sancar KAYA

**Affiliations:** 1 Department of Physical Medicine and Rehabilitation, Marmara University Faculty of Medicine, İstanbul, Turkey; 2 Department of Physical Medicine and Rehabilitation, Kırşehir Ahi Evran University Faculty of Medicine, Kırşehir, Turkey; 3 Department of Physical Medicine and Rehabilitation, İzzet Baysal Physical Treatment Training and Research Hospital, Bolu, Turkey; 4 Department of Pain Medicine, Ankara City Hospital, Ankara, Turkey

**Keywords:** Calcaneal spur, plantar fasciitis, size, type, extracorporeal shock wave therapy, pain

## Abstract

**Background/aim:**

The purpose of this study was to determine the effect of the presence, size, or type of calcaneal spurs on pain or the outcomes of extracorporeal shock wave therapy (ESWT) therapy in patients with plantar fasciitis.

**Materials and methods:**

Seventy-four patients with unilateral plantar fasciitis who had no pain in the contralateral foot, either currently or in the past, were included in the study. The length, base width, type, and presence of plantar calcaneal spurs in both heels of the patients were determined using radiography. A total of five sessions of ESWT (3 bar, 2000 shocks/session, 12 Hz frequency) with an interval of 3 days were performed on the painful sides of the patients. Symptom duration and numerical rating scale (NRS) scores were recorded pretreatment and 1 week and 12 weeks after treatment.

**Results:**

Spurs were detected in 85.1% of painful feet and 71.6% of painless feet, this difference was statistically significant (p = 0.046). There was no significant correlation between the type of the spurs and whether the foot was painful. Patients with spur sizes of >5 mm or with horizontal and hooked spurs had a higher NRS decrease than patients with spur sizes of ≤5 mm or with a vertical spur. Symptom duration, spur length, and base width were found to be correlated with pretreatment NRS scores.

**Conclusion:**

The presence and size of calcaneal spurs are associated with pain. However, it should be kept in mind that a high rate of spurs can also be found in painless feet, so spur is not the only factor that causes pain. Patients with a spur size of ≤5 mm or a vertical spur have less pain relief with ESWT.

## 1. Introduction

Plantar fasciitis (PF) is the most common cause of heel pain [1]. One in 10 people has PF at some time in their life [2, 3]. Diagnosis of PF is based on patient history, physical examination findings (plantar fascia tenderness, increased pain on palpation of the medial plantar calcaneal region), and risk factors. It is typical for patients with PF to feel heel pain and tension intensely after getting out of bed in the morning, which then improves with ambulation [4]. Radiography comes first in diagnostic imaging in patients with heel pain [3, 5]. Although magnetic resonance imaging (MRI) and ultrasonography (US) have advantages in directly evaluating the plantar fascia, radiographs allow the evaluation of conditions such as a tumor, fracture, and calcaneal spurs [3, 5–7].

Whether calcaneal spurs contribute to the symptoms is controversial. Some authors have described heel spur formation as a continuing process through constant traction from adjacent soft tissue, followed by chronic inflammation, periostitis, and osteogenesis of the spur, respectively [8]. Some investigators identify calcaneal spurs as a primary or contributing cause of heel pain rather than viewing it as the result of PF [9, 10]. Many patients with pain due to PF were found to have no calcaneal spurs [11]. In addition, a large population with calcaneal spurs has been identified with no heel pain [12, 13]. Although there are different results in different countries, calcaneal spurs are encountered at a high rate of up to 21% in the community. This rate reaches 55% over the age of 62 years [14].

The effectiveness of rest, activity modification, ice massage, oral analgesics, stretching techniques, physical therapy modalities, foot orthotics, night splinting, corticosteroid injections, extracorporeal shock wave therapy (ESWT), and plantar fasciotomy have been demonstrated in the treatment of PF [15]. ESWT, which has been shown to be an effective and safe treatment for PF, has been widely used recently [16, 17]. It was found that ESWT significantly reduced spur size and pain in patients with symptomatic calcaneal spurs [18].

The relation between calcaneal spurs with pain is not clear, and studies investigating the relationship between the size and type of spurs with pain or response to treatment are also limited. Ahmad et al. divided calcaneal spurs into three groups according to their type as horizontal, vertical, and hooked. They examined their types and sizes and their relationship with treatment outcomes consisting of weight-bearing, controlled ankle motion (CAM) walking boots, and a supervised rehabilitative physical therapy program. They found better improvement in horizontal and hook or large spurs than vertical or small spurs [3].

Although there is a study examining the effects of ESWT on radiological changes and pain in the calcaneal spur [19], we did not find any study examining the effect of spur size and spur type on clinical outcomes of ESWT. Despite the uncertainty of the presence, size, and type of calcaneal spurs, their effect on pain, and treatment response, there are various treatment alternatives for calcaneal spurs and some physicians request radiography to investigate calcaneal spurs in patients with heel pain [18, 20]. Accordingly, it is important to reveal the relationship between the presence, size, and type of calcaneal spurs with pain and treatment outcomes. This study aimed to reveal whether calcaneal spurs were associated with pain and to investigate the relationship between spur type, size, and ESWT treatment outcomes in patients with PF.

## 2. Materials and methods

### 2.1. Study population

Seventy-eight patients who were diagnosed as having unilateral PF were evaluated for inclusion in this prospective study. Since there is no study similar to ours, the sample size was calculated using the standard effect sizes recommended by Cohen [21], which are widely used in the literature (Effect size d = 0.5, Power (1-β err prob) = 0.90). The sample size of the study was calculated with the G-Power package program (Version 3.1.9.6; Universitat Kiel, Germany) using priori power analysis.

The study was conducted at Kırşehir Ahi Evran University Hospital between June 2020 and June 2021. The presenting symptom of these patients was plantar heel pain. Patients who were diagnosed as having PF by a physiatrist based on patient history, risk factors, and physical examination were recruited for the study. All patients were evaluated by the same physician (FAK) in terms of diagnosis. All patients had plantar heel pain, which is typical of PF, which was felt strongly when getting out of bed in the morning and then subsided with ambulation. In addition, a detailed physical examination was performed on all patients. Other diseases in the differential diagnosis (e.g., lumbar discal hernia, neuroma, fracture) were evaluated comprehensively. Other specific tests were performed to rule out these disorders.

The inclusion criteria were age of 18–65 years, being diagnosed as having unilateral PF and approving to participate in the study. The exclusion criteria were having a history of heel pain in the opposite foot currently or in the past, having received treatment for PF in the last 6 months, a history of foot/heel fracture, surgery, inflammatory rheumatic disease, having a disease that might affect foot function (e.g., lumbar disc hernia, neuroma).

### 2.2. Outcome measures

**Figure 1:**
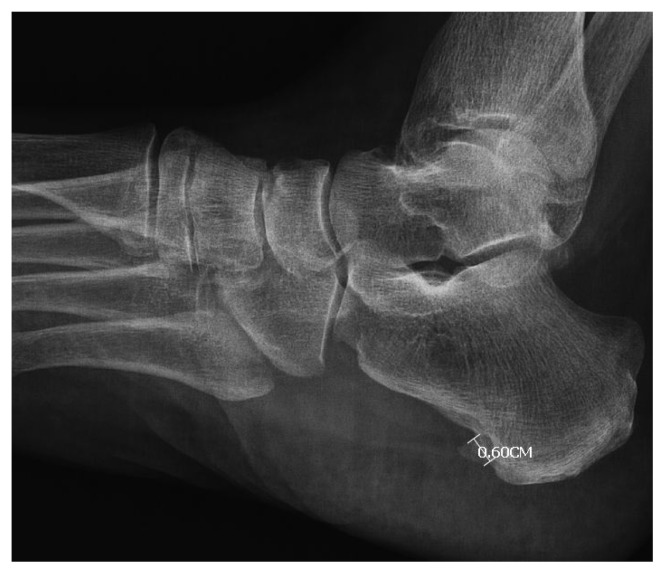
Calcaneal spur base width measurement.

**Figure 2:**
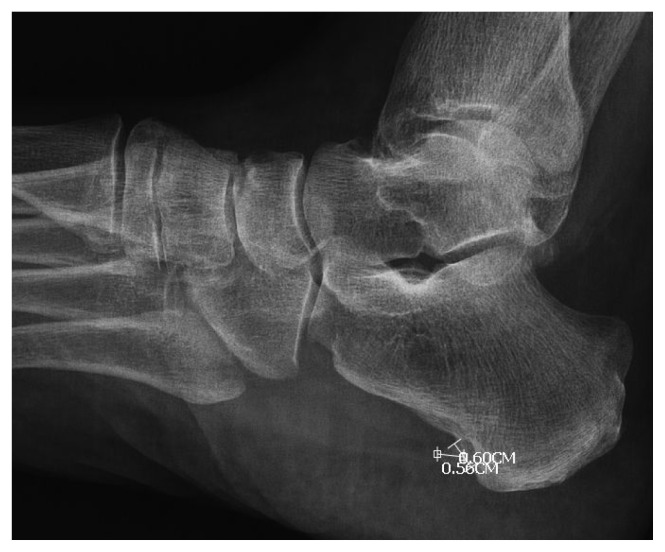
Calcaneal spur length measurement.

The age, sex, height, weight, BMI, painful side, and the duration of symptoms of the patients were recorded. Perceived pain intensity was evaluated using a numerical rating scale (NRS) pretreatment and 1 week and 12 weeks after treatment. In NRS measurements, the patients were asked to give pain scores between 0 and 10 depending on the severity of the pain. High scores meant more severe pain [22]. On lateral calcaneal radiographs, computer-aided linear measurements were recorded for spur length (mm) from tip to base as defined by a line demarcating the calcaneal border for both feet as described by Johal [10]. In addition, the type of the spur and the width of the base were recorded (Figures 1 and 2). The calcaneal spurs were divided into groups as horizontal, vertical, and hooked according to their types as in the study of Ahmad et al. Vertical spurs run perpendicular to the base of the calcaneus. Horizontal spurs run relatively parallel to the base of the calcaneus. The tip of hook spurs forms a hook-like structure. In addition, in this study, as in the study of Ahmad et al., spurs were divided into two groups according to their size: spurs >5 mm and spurs ≤5 mm [3]. All measurements were performed by an independent researcher (YE) who was blinded to the patients’ clinical data.

### 2.3. Ethical approval

The protocol was performed in accordance with the ethical standards laid down in the 1975 Declaration of Helsinki and approved by Kırşehir Ahi Evran University Medical Faculty Clinical Research Ethics Committee (Date: 10.06.2020, No: 2020-08 / 56). All patients gave written informed consent.

### 2.4. Intervention

A total of five sessions of ESWT with an interval of 3 days were performed on the painful sides of the patients (3 bar, 2000 shocks/session, 12 Hz frequency). The treatment was performed in the supine position using a Modus ESWT Touch Shock Waves device (origin: Turkey). The five most sensitive and painful points in the plantar fascia area were determined and 400 beats were given to each point. No analgesic or local anesthetic medication was administered before or during the treatment. The same physiotherapist performed the ESWT on all patients. Patients did not receive any treatment other than ESWT.

### 2.5. Statistical analysis

The normality assumption for quantitative variables was tested using the Kolmogorov–Smirnov and Shapiro–Wilk tests. Explanatory statistics of the variables are given as mean ± standard deviation, median (min–max), and frequencies n (%). For the univariate analysis of the variables in the study, the Mann–Whitney U, Kruskal–Wallis, and Wilcoxon tests were used according to the variable type and the assumptions. The differences between the categories of the observations in the categorical variables were tested using the chi-squared test. Relationships between categorical data were tested using the Fisher–Freeman–Halton exact test. Relationships between quantitative variables were analyzed using Spearman’s correlation analysis. In all statistical analyses, cases with a p-value below 0.05 were interpreted as statistically significant. Statistical analysis of the study was performed using SPSS v. 21.0 software for Windows (IBM SPSS Armonk, NY: IBM Corp., USA).

## 3. Results

Seventy-eight patients were examined to reach the required sample size calculated as 74. Two patients refused to participate in the study. Another two patients were excluded because corticosteroids were administered by physicians from another center. As a result, 74 patients were included in the study.

The mean age of the patients was 47.7 ± 10.06 years. The mean duration of symptoms was 10.8 ± 11.02 months. The general characteristics of the participants are summarized in Table 1.

In radiologic examinations, no statistically significant difference was found between the painful sides and the nonpainful sides in terms of spur length and base width (p > 0.05). The spur types in the foot radiographs were divided into four groups: 0 = absent, 1 = hooked, 2 = horizontal, and 3 = vertical [3]. There was no significant correlation between the type of spur and whether the foot was painful (p > 0.05). There was a statistically higher number of spurs in painful feet than in painless feet (p = 0.046) (Table 2).

The median pretreatment NRS value was 8 and was determined as 5 in the 1st week after treatment. At the 12th-week evaluation, the median NRS was found as 3.5. The differences between the pretreatment, 1st-week, and 12th-week NRS values were found to be statistically significant (Table 3).

The relationships between the pretreatment, 1st-week, and 12th-week NRS scores of the patients and sex, spur type, and spur length are shown in Table 3.

The patients were divided into two groups according to the length of the spur on the painful side as ≤5 mm and >5mm [3].

The pretreatment NRS scores of the patients with a spur length of >5 mm on the painful side were higher than those of the ≤5 mm group (p < 0.05). There was no significant difference between the 1st- and 12th-week NRS scores between the two groups (p > 0.05). In the ≤5 mm group, a significant decrease in NRS scores was observed at the 1st and 12th weeks compared with pretreatment (p < 0.01). The difference between the 12th-week NRS scores and the 1st-week NRS scores in the ≤5 mm group was not statistically significant. In the group with spurs > 5 mm, a significant decrease in NRS scores was found at the 1st and 12th weeks compared with pretreatment (p < 0.01). However, unlike the ≤5 mm group, the 12th-week NRS scores were lower than the 1st-week NRS scores in the group with spurs > 5 mm. In other words, the downward trend in NRS scores continued until the 12th week.

The relationship between symptom duration and pretreatment NRS scores was also positively significant (Rho = 0.286, p < 0.05). The relationship between pretreatment NRS scores with the length (Rho = 0.322, p < 0.01) and base width (Rho = 0.261, p < 0.05) of spurs in the painful feet was also positive and significant (Table 4).

The relationship between the symptom duration of the patients and the spur length on the painful side was positive and statistically significant (Rho = 0.236, p < 0.05). Among the patients with spurs on the painful side, 23 (37%) patients had spur lengths shorter than those on the painless side.

## 4. Discussion

In this study, it was determined that PF was associated with the presence of spurs, and spur length and base width were associated with pain level, but spurs were also detected in many painless feet. No relationship was found between spur type and pretreatment pain levels. However, vertical spurs were associated with worse treatment outcomes than the other types. Patients with longer spurs had more pain relief with ESWT than those with smaller spurs.

In a study by Kuyucu et al., which did not include a control group, the authors found 80% of spurs in patients with PF. They showed that the length of calcaneal spurs was correlated with symptom duration, pain, and function [4]. In their retrospective study, Johal et al. found that 89% of patients with PF had spurs and significantly more spurs were present than in those without PF [10]. In the present prospective study, 85% of the sides with PF were found to have calcaneal spurs. This ratio is close to those found in other studies. Similarly, a significantly higher rate of plantar calcaneal spurs was detected in painful heels compared with painless heels. Detection of more spurs in patients with PF may be due to the similarity of many predisposing factors of PF and spurs [14, 23]. Moreover, some authors explain the calcaneal spur as a chronic consequence of PF [8]. This may explain the increase in calcaneal spurs in PF. Further studies are needed to confirm these possibilities. The asymptomatic contralateral foot of the same patient was evaluated as the control group. In this way, intrinsic and extrinsic factors such as weight, standing time, additional diseases, and shoes will be equalized between both groups. Therefore, the effect of spurs could be evaluated more fairly.

In the present study, a significant correlation was found between spur length and pain level, and symptom duration. Some authors have described heel spur formation as a continuing process through constant traction from adjacent soft tissue, followed by chronic inflammation, periostitis, and osteogenesis of the spur, respectively. Considering that this process will take time, it can be thought that PF, which lasts longer and more severely, contributes more to the development of spurs over time. This may explain the positive correlation of spur length with pain level and symptom duration in our study [8]. Unlike other studies, we found that the base width of the spur was also related to the level of pain. This is the first study to examine the relationship between spur base width and pain. In addition, in the present study, 71.6% of the patients had spurs on the painless side and 14.9% of the patients had no spurs on the painful side. Moreover, it was found that 37% of the patients with spurs on the painful side had a smaller spur size than the spurs in the painless foot. These results can be interpreted as that the presence of spurs affects pain, but it is not always the primary factor. Already, in some previous studies, it has been shown that patients with no heel pain or PF have a high rate of calcaneal spurs, and a significant number of patients with PF do not have spurs [11–13].

Studies investigating the relationship between spur type and pain level are limited. Ahmad et al. showed that spur types were not associated with pain and function [3]. Similarly, in the present study, we found that the type of spur did not affect pain. Functional evaluation was not performed in this study. Only one study by Ahmad et al. tried to answer the question of whether the presence, size, or type of spurs affected treatment outcomes. They retrospectively examined the effect of CAM walking boots and rehabilitative physiotherapy [3]. They found greater improvement in horizontal and hook spurs than vertical spurs with CAM walking boots and rehabilitative physiotherapy. They also observed superior improvement in spurs > 5 mm compared with spurs < 5 mm [3]. Similarly, in the present study, it was observed that spurs > 5 mm showed better improvement than spurs ≤ 5 mm. In addition, in this study, similar to the study of Ahmad et al., patients with horizontal and hooked spurs had more pain relief than those with vertical spurs. The worse prognosis of vertical spurs may be due to the fact that the spur is more perpendicular to the plantar fascia, thus irritating the plantar fascia more [3]. Zhou et al. examined patients with calcaneal spur and PF endoscopically. They found that patients with calcaneal spurs located within the plantar fascia had a more severe grade of plantar fasciitis than patients with calcaneal spurs located superior to the plantar fascia insertion [24]. The differences in the types of spurs are associated with the differences in the structures from which the spurs originate. The majority of spurs originate from the medial process of the tuberosity, but in some patients, they may also arise from the lateral processes and sulcus [14]. Therefore, the difference in treatment results between the types may also be due to the differences in the structures from which the spurs originate. There is evidence that larger spurs show a greater level of cortical thickening compared with smaller spurs [14]. However, it is not clear whether a better prognosis of larger spurs is due to the difference in cortical thickness. More study is needed on this subject.

Hayta et al. reported that ESWT reduced calcaneal spur lengths and pain in patients with symptomatic calcaneal spurs [18]. Mishra et al. showed that ESWT and methylprednisolone injections were both effective on pain in PF, but ESWT was more effective than methylprednisolone injections [16]. Yalçın et al. examined the radiological change in spurs caused by ESWT. They found that the radiological changes that occurred after ESWT were not related to clinical outcomes [19]. Although there are studies showing the effect of ESWT on PF and calcaneal spur, we have not found any studies examining the effect of spur presence, size, or type on treatment outcomes of ESWT [25, 26].

### 4.1. Limitations

Although the sample size was calculated before the study, the small number of participants is one of the limitations of this study. More patient participation and longer follow-up would have made the study more valuable. In addition, the fact that the distribution of symptom duration had a wide range may have affected the results of the study. The lack of MRI, USG, and functional evaluations can be counted as other limitations. However, this study is the first study to compare the PF side of the same patient with the asymptomatic side without a history of PF. It is also the first study to examine the effect of spur size or type on ESWT outcomes and to evaluate calcaneal spur base width.

### 4.2. Conclusion

In this study, it was determined that PF was associated with the presence of spurs, and spur length and base width were associated with pain level. Spurs were also detected in many painless feet, so although the spur affects the pain, it is not the only source of the pain. Patients with longer spurs had more pain relief with ESWT than those with smaller spurs. No relationship was found between spur type and pretreatment pain levels, but vertical spurs were associated with worse treatment outcomes than the other types.

## Figures and Tables

**Table 1:** General characteristics of the participants.

	Mean ± SD	Min–max
Age (years)	47.7 ± 10.06	25–65
BMI (kg/m^2^)	30.38 ± 5.27	18.59–49.90
Symptom duration (months)	10.8 ± 11.02	1–48
Standing time (hours)	8.22 ± 2.44	4–14

BMI: Body mass index, SD: standard deviation, Min: minimum, Max: maximum

**Table 2:** Presence, length, and base width and type of spur on painful and painless sides.

		Painful heels (n = 74)	Painless heels (n = 74)	p
		Median (min–max)	Median (min–max)	
	Spur length (mm)	4.2 (0.0–16.30)	3.25 (0.0–11.0)	0.253^a^
	Spur base width (mm)	5.4 (0.0–17.30)	4.90 (0.0–11.50)	0.138^a^
Presence of spur				
	Number of feet without spurs	11 (14.9%)	21 (28.4%)	**0.046** a
	Number of feet with spurs	63 (85.1%)	53 (71.6%)	
Type of spur				
	0 (absent)	11 (14.9)	21 (28.4)	0.053^a^
	1 (hooked)	14 (18.9)	11 (14.9)	
	2 (horizontal)	45 (60.8)	42 (56.8)	
	3 (vertical)	4 (5.4)	-	
	***p***	***<0.001*** b	***<0.001*** b	

Min: minimum, max: maximum

a p-value of statistics between groups

b p-value of the comparison of spur types in the same group

**Table 3:** Relationship between pretreatment, 1st-week, and 12th-week NRS scores of the patients and sex, spur type, and spur length.

		Pretreatment NRS score	1st-week NRS score	12th-week NRS score	P^Pre-1st week^	P^Pre-12th week^	P^1st-12th week^
		Median (min–max)	Median (min–max)	Median (min–max)			
	All painful feet	8 (5.0–10.0)	5 (1.0–10.0)	3.5 (0.0–10.0)	**<0.001**	**<0.001**	**0.004**
Sex	Male	7 (6.0–9.0)	4 (1.0–9.0)	3 (0.0–8.0)	**<0.001**	**<0.001**	0.084
	Female	8 (5.0–10.0)	5 (1.0–10.0)	4 (0.0–10.0)	**<0.001**	**<0.001**	**0.022**
	***P*** ^a^	*0.077*	*0.161*	*0.357*			
Spur type							
	0 (absent)	7 (6.0–9.0)	4 (2.0–9.0)	6 (0.0–9.0)	**0.024**	**0.035**	0.569
	1 (hooked)	8 (5.0–10.0)	5.5 (2.0–9.0)	3 (0.0–10.0)	**0.002**	**0.004**	0.073
	2 (horizontal)	8 (5.0–10.0)	5 (1.0–10.0)	3 (0.0–9.0)	**<0.001**	**<0.001**	**0.039**
	3 (vertical)	8 (7.0–9.0)	4.5 (3.0–7.0)	3 (0.0–7.0)	0.068	0.109	0.357
	***P*** ^b^	0.051	0.365	0.904			
Spur length							
	≤5 mm (n=48)	8 (5.0–10.0)	5 (1.0–9.0)	4.5 (0.0–10.0)	***<0.001***	***<0.001***	*0.096*
	>5 mm (n=26)	8.5 (7.0–10.0)	5 (1.0–10.0)	3 (0.0–9.0)	***<0.001***	***<0.001***	***0.006***
	***P*** ^c^	***0.022***	*0.362*	*0.117*			

NRS: Numerical rating scale, min: minimum, max: maximum, P^Pre -1st week^: Comparisons of pretreatment NRS and 1st-week NRS scores in the same group; P^Pre-12th week^ : Comparisons of pretreatment NRS and 12th-week NRS scores in the same group; P^1st-12th week^: Comparisons of 1st-week NRS and 12th-week NRS scores in the same group; P^a^: Comparisons of NRS scores between male and female; P^b^: Comparisons of NRS scores between spur types; P^c^: Comparisons of NRS scores of patients with spurs ≤5 mm and patients with spurs >5 mm.

**Table 4:** Correlation table between NRS scores with age, BMI, symptom duration, spur length and base width in painful feet.

	Pretreatment NRS	1st-week NRS	12th-week NRS
**Age**	0.119	−0.022	−0.238
**BMI**	0.088	−0.038	−0.207
**Symptom duration**	0.286*	−0.140	−0.047
**Length of spur**	0.322**	−0.031	−0.132
**Base width of spur**	0.261*	−0.034	−0.132

NRS: Numerical rating scale, BMI: Body mass index,

* p < 0.05;

** p < 0.01
